# Efficacy of lisdexamfetamine dimesylate in children with attention-deficit/hyperactivity disorder previously treated with methylphenidate: a post hoc analysis

**DOI:** 10.1186/1753-2000-5-35

**Published:** 2011-11-04

**Authors:** Rakesh Jain, Thomas Babcock, Teodor Burtea, Bryan Dirks, Ben Adeyi, Brian Scheckner, Robert Lasser

**Affiliations:** 1 Department of Psychiatry and Behavioral Sciences, University of Texas Medical School, Houston, Texas, and R/D Clinical Research, Inc, Lake Jackson, Texas, USA; 2 Shire Development Inc., Wayne, Pennsylvania, USA; 3 Formerly of Shire Canada Inc., Saint-Laurent, QC, Canada

**Keywords:** Attention-deficit/hyperactivity disorder (ADHD), lisdexamfetamine dimesylate (LDX), methylphenidate, children, efficacy

## Abstract

**Background:**

Attention-deficit/hyperactivity disorder (ADHD) is a common neurobehavioral psychiatric disorder that afflicts children, with a reported prevalence of 2.4% to 19.8% worldwide. Stimulants (methylphenidate [MPH] and amphetamine) are considered first-line ADHD pharmacotherapy. MPH is a catecholamine reuptake inhibitor, whereas amphetamines have additional presynaptic activity. Although MPH and amphetamine can effectively manage ADHD symptoms in most pediatric patients, many still fail to respond optimally to either. After administration, the prodrug stimulant lisdexamfetamine dimesylate (LDX) is converted to l-lysine and therapeutically active d-amphetamine in the blood. The objective of this study was to evaluate the clinical efficacy of LDX in children with ADHD who remained symptomatic (ie, nonremitters; ADHD Rating Scale IV [ADHD-RS-IV] total score > 18) on MPH therapy prior to enrollment in a 4-week placebo-controlled LDX trial, compared with the overall population.

**Methods:**

In this post hoc analysis of data from a multicenter, randomized, double-blind, forced-dose titration study, we evaluated the clinical efficacy of LDX in children aged 6-12 years with and without prior MPH treatment at screening. ADHD symptoms were assessed using the ADHD-RS-IV scale, Conners' Parent Rating Scale-Revised short form (CPRS-R), and Clinical Global Impressions-Improvement scale, at screening, baseline, and endpoint. ADHD-RS-IV total and CPRS-R ADHD Index scores were summarized as mean (SD). Clinical response for the subgroup analysis was defined as a ≥ 30% reduction from baseline in ADHD-RS-IV score and a CGI-I score of 1 or 2. Dunnett test was used to compare change from baseline in all groups. Number needed to treat to achieve one clinical responder or one symptomatic remitter was calculated as the reciprocal of the difference in their proportions on active treatment and placebo at endpoint.

**Results:**

Of 290 randomized participants enrolled, 28 received MPH therapy at screening, of which 26 remained symptomatic (ADHD-RS-IV > 18). ADHD-RS-IV total scores, changes from baseline, clinical responsiveness, and rates of symptomatic remission in this subgroup were comparable to the overall population. The safety and tolerability profiles for LDX were comparable to other stimulants currently available.

**Conclusion:**

In this analysis, children with significant clinical ADHD symptoms despite MPH treatment improved during treatment with LDX and experienced similar improvements in their symptoms as the overall study population.

**Trial Registration:**

ClinicalTrials.gov: NCT00556296

## Background

Attention-deficit/hyperactivity disorder (ADHD) is one of the most common neurobehavioral psychiatric disorders that afflicts children [1], with a reported prevalence of 2.4% to 19.8% worldwide [2] using the criteria from the *Diagnostic and Statistical Manual of Mental Disorders*, *Fourth Edition *(DSM-IV) from the American Psychiatric Association [3]. Two Canadian studies of children and adolescents, using earlier diagnostic criteria to examine ADHD prevalence, estimated a prevalence of 6.3% in an Ontario study of participants (aged 4 to 16 years) [4], and 3.3% to 8.9% in a comparable population (aged 6 to 14 years) in Quebec [5].

Stimulants have long been used to treat ADHD symptoms. The Texas Consensus Conference Panel on Pharmacotherapy of Childhood ADHD algorithm [6] considered psychostimulants as first-line pharmacotherapy treatments for ADHD; However, the Canadian ADHD Resource Alliance (CADDRA) guidelines consider long-acting stimulants and atomoxetine as first-line agents in the management of ADHD [7]. The stimulant types most commonly used in ADHD treatment are methylphenidate (MPH) and amphetamine. These have similar subjective effects [8] yet differ in their mechanisms of action--MPH is a dopamine and norepinephrine reuptake inhibitor, while amphetamines have additional presynaptic activity--stimulating the release of dopamine, norepinephrine, and serotonin [9]. Although both are considered efficacious, a meta-analysis of 23 studies comparing the efficacy of immediate-release (IR) formulations of MPH and amphetamine in the treatment of children with ADHD revealed small but statistically significant differences in favor of amphetamine [10]. A comparative review of controlled crossover studies [11] found that clinical response rates for IR formulations of MPH and amphetamine ranged from 57% to 68% and 69% to 77%, respectively. The review also estimated that 87% to 92% participants respond to at least one of these stimulants. However, although MPH and amphetamine can effectively manage ADHD symptoms in most pediatric patients, many patients still fail to respond optimally to either.

Lisdexamfetamine dimesylate (LDX; Vyvanse^®^) is a prodrug stimulant with a novel delivery mechanism, approved in Canada [12] and the United States [13] for the treatment of ADHD in children 6 to 12 years of age, adolescents aged 13 to 17 years, and adults. LDX is a therapeutically inactive molecule. LDX is converted, primarily in the blood, to l-lysine and therapeutically active d-amphetamine [14]. In Canada, the approved dosages range from 20 to 60 mg capsules for once daily oral administration and in the United States from 20 to 70 mg also once daily [12,13].

LDX has been shown to be effective from 1.5 to 13 hours postdose in children [15], and from 2 to 14 hours postdose in adults [16]. In a randomized controlled trial (RCT), LDX was associated with improvements in clinical symptoms of ADHD in children while maintaining a safety profile similar to other stimulant medications [17].

In this post hoc analysis from the RCT, the efficacy of LDX in a subset of children, who had significant ADHD symptoms at study enrollment despite receiving MPH treatment, was evaluated to determine clinical response to LDX therapy in these study participants. Based on previous findings that some patients fail to achieve optimal response to either MPH or amphetamine, children who were previously treated with MPH and continue to have ADHD symptoms may be responsive to amphetamine-based ADHD treatment.

## Methods

The methods used in this study for the overall study population have been described previously [17]. This was a multicenter, randomized, double-blind, forced-dose titration, parallel-group study, conducted in accordance with the Guideline for Good Clinical Practice from the World Health Organization and the Declaration of Helsinki and its amendments.

### Participants

Biederman et al previously described full inclusion/exclusion criteria [17]. Briefly, children aged 6 to 12 years who met DSM-IV-TR criteria for a primary diagnosis of ADHD [18] and had a ADHD Rating Scale IV (ADHD-RS-IV) [19,20] score of ≥ 28 at baseline after washout were eligible for inclusion, regardless of medication used for ADHD at screening.

### Study Design

The study comprised a 1-week screening period; a 1-week washout period of prior psychoactive medications; and 4-weeks of double-blind treatment. During screening, participants received an initial ADHD-RS-IV evaluation. Participants receiving medication for ADHD at enrollment were allowed to continue their medication during the screening evaluation. After screening, the parents/caregivers of eligible participants were instructed to discontinue their prior ADHD medications, if they had not already done so.

Baseline assessments were made after the 1-week washout. Participants were randomized in a 1:1:1:1 ratio (using a block-randomization schedule) to receive double-blind, oral administration of LDX 30 mg/day for 4 weeks, 50 mg/day (30 mg/day for week 1, 50 mg/day for weeks 2 to 4), 70 mg/day (30 mg/day for week 1, 50 mg/day for week 2, 70 mg/day for weeks 3 and 4), or placebo for 4 weeks.

### Efficacy Outcome Measures

The primary efficacy outcome was the change in mean ADHD-RS-IV total score from baseline to treatment endpoint, defined as the last postrandomization week for which a score was obtained. ADHD-RS-IV total score assessments were based on investigator interviews with the caregiver and child regarding symptom severity during the preceding week.

Secondary efficacy measures included ADHD-RS-IV total scores at screening, baseline, and endpoint; percent change in ADHD-RS-IV total score; the Conners' Parent Rating Scale-Revised (CPRS-R: Short Form) [21]; and the investigator-rated Clinical Global Impressions (CGI) scale [22]. The CGI-Severity (CGI-S) assessment was conducted at the baseline visit and the CGI-Improvement (CGI-I) assessment was conducted at subsequent visits.

Efficacy was assessed in the overall efficacy population, all participants who had ADHD-RS-IV scores recorded at baseline and at least one other postrandomization time point.

### Post Hoc Efficacy Analyses

This post hoc efficacy analysis assessed treatment effects of LDX and placebo in participants receiving MPH prior to entering the present study, who had available screening data and significant ADHD symptoms prior to discontinuing their MPH regimen. Efficacy was further evaluated according to mean daily MPH dose received (≥ 1 mg/kg vs < 1 mg/kg) during prior treatment.

Rates of symptomatic remission and clinical response were evaluated throughout the study in participants receiving prior MPH therapy and the efficacy population.

Steele et al [23] suggested that treatment response be considered as an improvement in symptom scores from baseline of 25% to 30%. However, reductions from baseline do not take into account potential differences in baseline severity of disease. Participants with severe symptoms at baseline may be considered responders but still exhibit symptoms. Hence, a clinical response definition that includes a percent reduction in symptoms and a measure of global clinical improvement, such as the CGI-I, may be a better measure of clinical response to treatment. Moreover, other studies have shown that a 1-level change on the CGI-I was consistent with an estimated 10- to 15-point or 25% to 30% change from baseline in ADHD-RS-IV total score [24].

In the primary analysis [17], Biederman et al reported on the ADHD-RS-IV (primary outcome measure) and CGI-I (secondary outcome measure) as continuous measures. In this present analysis, clinical response to LDX treatment was defined as a dual criteria of ≥ 30% reduction in ADHD-RS-IV total score from baseline and a CGI-I score of 1 or 2 at endpoint based on data from previous reports defining response [23,25]; symptomatic remission was defined as ADHD-RS-IV total score of ≤ 18 [26]. Conversely, nonremitters on prior MPH were defined as participants with an ADHD-RS-IV total score > 18 while receiving MPH prior to entering the study. Number-needed-to-treat (NNT) for 1 participant to achieve a therapeutic clinical response or symptomatic remission at treatment endpoint was calculated to translate the efficacy data into more clinically meaningful terms.

### Safety Assessments

Safety assessments, in enrolled participants who received at least 1 dose of study medication, have been reported previously [17]. Briefly, these included adverse events (AEs), electrocardiograms (ECGs), blood pressure (BP), heart rate, and laboratory assessments. Treatment-emergent AEs (TEAEs) were coded using the *Medical Dictionary for Regulatory Activities *version 7.1 [27]. TEAEs referred to events with onset after the first date of treatment and no later than 3 days following termination of treatment. No separate assessments were performed in nonremitters on prior MPH due to low sample numbers and no reason to expect differences in safety/tolerability in these participants.

### Statistical Analyses

ADHD-RS-IV total and CPRS-R ADHD Index scores were summarized as mean (standard deviation [SD]). Mean change in ADHD-RS-IV total score for the overall population was assessed using 2-way analysis of covariance. Dunnett test for multiple mean comparisons with least-squares adjustment was used to compare change from baseline in the 3 active treatment groups versus placebo. NNT to achieve 1 clinical responder or 1 symptomatic remitter was calculated as the reciprocal of the difference in proportions of clinical responders or symptomatic remitters on active treatment and placebo at treatment endpoint.

## Results

### Participant Demographics and Disposition

In total, 297 children were enrolled at 40 study sites in the United States, of which 7 children discontinued prior to randomization, and 290 were randomized to receive LDX (n = 218) or placebo (n = 72). Of these, 285 had a postrandomization symptom assessment and were included in the efficacy population. Full demographic data for this population have been previously reported [17].

Of the 290 randomized participants, 28 were receiving MPH treatment at screening and 26 of these were classified as nonremitters on prior MPH at the screening visit, prior to randomization (Table 1). Median age was 9 years and 11/26 (42.3%) female and 15/26 (57.7%) male participants were included. Prior treatment for 19 (73.1%) participants was osmotic, controlled-release MPH (OROS MPH), alone or in combination with another ADHD medication (1 participant in combination with IR dex-MPH [d-MPH], 1 with IR mixed amphetamine salts); 2 (7.7%) participants received prior treatment with extended release (ER) MPH; 3 (7.7%) participants received prior treatment with IR MPH; 1 (3.8%) participant was previously treated with sustained release MPH (SR MPH); 1 (3.8%) participant was prior treated with MPH controlled delivery (MPH CD) (Table 1). Sixteen participants (61.5%) received an average daily dose of ≥ 1 mg/kg MPH, and 10 (38.5%) an average daily dose of < 1 mg/kg MPH.

**Table 1:** Baseline Demographic and Clinical Characteristics of Randomized Participants Classified as Nonremitters During Prior MPH Treatment

Participant	Age (years)	Sex	Weight (kg)	Medication	Total Daily Dose (mg/day)	Average Daily Dose (mg/kg)	Screening ADHD-RS-IV Total Score
1	6	F	22.68	OROS MPH*	≥ 30	≥ 1.0	50

2	7	M	29.48	ER MPH	20	< 1.0	20

3	7	F	44.45	OROS MPH	36	< 1.0	28

4	8	M	44.36	OROS MPH	18	< 1.0	38

5	8	M	26.31	OROS MPH	54	≥ 1.0	38

6	8	M	33.57	OROS MPH; IR dMPH	54; 2.5	≥ 1.0	50

7	8	M	41.28	OROS MPH	18	< 1.0	43

8	8	F	43.68	IR MPH	30	< 1.0	50

9	9	M	29.03	ER MPH	20	< 1.0	29

10	9	M	26.76	OROS MPH	54	≥ 1.0	39

11	9	M	31.75	MPH CD	40	≥ 1.0	29

12	9	M	26.31	OROS MPH	27	≥ 1.0	45

13	9	F	28.12	SR MPH	20	< 1.0	40

14	9	F	25.18	IR MPH*	≥ 50	≥ 1.0	34

15	9	F	24.90	OROS MPH	27	≥ 1.0	20

16	10	M	39.01	OROS MPH	54	≥ 1.0	23

17	10	F	43.68	OROS MPH^†^	36	< 1.0	22

18	10	F	27.67	OROS MPH	54	≥ 1.0	37

19	10	F	29.03	IR MPH	50	≥ 1.0	44

20	11	M	45.36	OROS MPH	72	≥ 1.0	35

21	11	M	45.36	OROS MPH	36	< 1.0	41

22	12	M	39.46	OROS MPH	54	≥ 1.0	45

23	12	M	34.02	OROS MPH	18	< 1.0	45

24	12	M	33.57	OROS MPH	54	≥ 1.0	44

25	12	F	34.02	OROS MPH	36	≥ 1.0	51

26	12	F	26.76	OROS MPH	54	≥ 1.0	25

ADHD-RS-IV = Attention-Deficit/Hyperactivity Disorder Rating Scale IV; dMPH = dexmethylphenidate; ER = extended-release; IR = immediate-release; MPH = methylphenidate; CD = controlled delivery; OROS = osmotic-release oral system; SR = sustained-release.

*Exact dose of treatment for these participants could not be determined; ^†^Participant was also receiving 40 mg/d of IR mixed amphetamine salts although this was not included in the calculation of MPH dose.

### Changes in ADHD-RS-IV Total Scores

**Figure 1:**
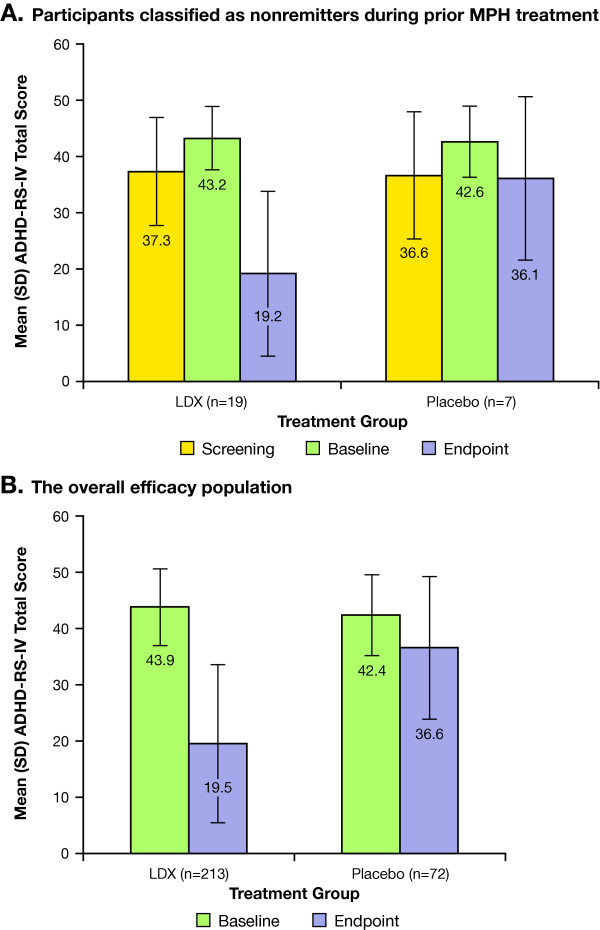
**ADHD-RS-IV total scores in (A) nonremitters during prior MPH treatment; and (B) the overall efficacy population**.

Mean (SD) screening, baseline, and endpoint ADHD-RS-IV total scores for nonremitters during prior MPH treatment, nonremitters stratified according to prior MPH dosage received, and overall efficacy population are shown in Figure 1.

**Figure 2:**
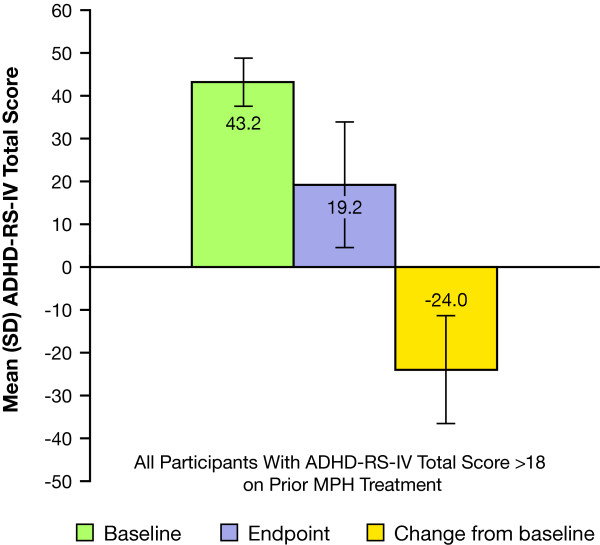
**ADHD-RS-IV total scores in prior MPH participants receiving LDX and classified as nonremitters**.

The mean (SD) change in ADHD-RS-IV total score from baseline with LDX treatment was -24.0 (12.56) (Figure 2), corresponding to a mean (SD) percentage reduction of 57 (29.9%) in the 19 nonremitters on prior MPH treatment. The mean (95% confidence interval [CI]) placebo-adjusted ADHD-RS-IV total score reduction for this group was -17.6 (-29.65, -5.49; *P *= .0063).

### Changes in ADHD-RS-IV and CGI-I Scores: Remitters and Responders

**Figure 3:**
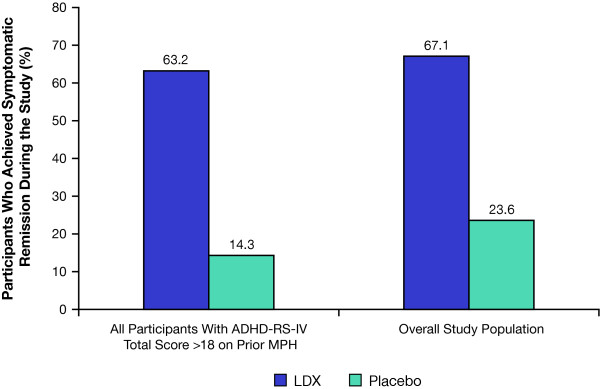
Percentage of symptomatic remitters* during the study. *Symptomatic remitters = participants who achieved ADHD-RS-IV total scores ≤ 18.

Of the 26 nonremitters on prior MPH at screening, 12 (63.2%) participants receiving LDX and 1 (14.3%) receiving placebo were classified as remitters during the study (Figure 3). Similar patterns of symptomatic remission with LDX treatment were observed in the overall efficacy population. As well, patterns of symptomatic remission were similar with placebo treatment in the overall efficacy population and in nonremitters on prior MPH. The NNT (95% CI) to achieve symptomatic remission with LDX at treatment endpoint was 2.0 (1.21, 6.63) in nonremitters and 2.1 (1.74, 2.72) in the overall study population.

**Figure 4:**
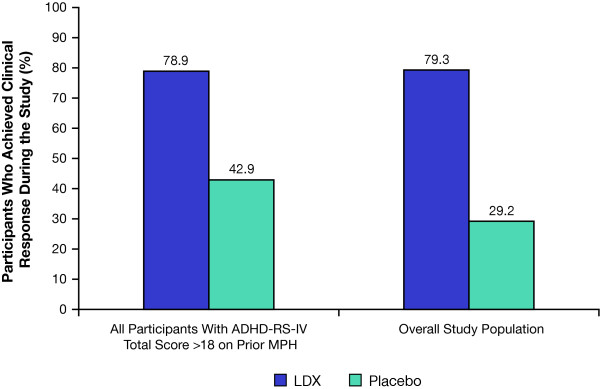
Percentage of clinical responders* during the study. *Clinical responders = participants who achieved ≥ 30% reduction in ADHD-RS-IV total scores from baseline and CGI-I scores of 1 or 2.

Of nonremitters on prior MPH, clinical response was achieved in 15 (78.9%) treated with LDX and 3 (42.9%) treated with placebo, respectively. In the overall efficacy population, 169 (79.3%) treated with LDX and 21 (29.2%) treated with placebo achieved clinical response (Figure 4). Of the 169 LDX clinical responders, 54 (32.0%) received 30 mg/d LDX, 55 (32.5%) received 50 mg/d LDX, and 60 (35.5%) received 70 mg/d LDX. NNT (95% CI) to achieve clinical response with LDX at treatment endpoint was 2.0 (1.21, 6.63) in nonremitters on prior MPH, versus 1.8 (1.51, 2.22) in the overall population.

### Changes in CPRS-R ADHD Index Scores

**Figure 5:**
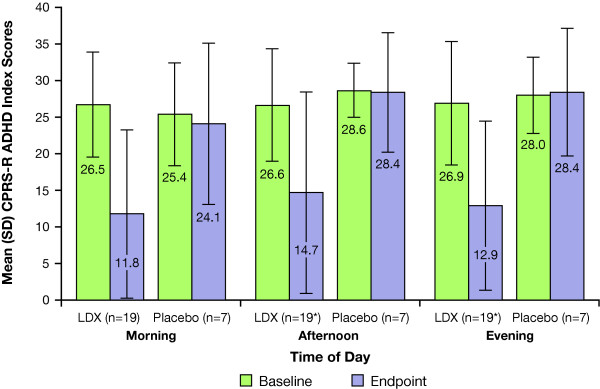
**CPRS-R ADHD index scores in prior MPH participants with ADHD-RS-IV total scores > 18 at screening**. *Data available for 18 participants receiving LDX at baseline, afternoon, and evening time points.

Mean (SD) morning, afternoon, and evening CPRS-R ADHD index scores at baseline and endpoint in nonremitters on prior MPH are shown in Figure 5. The mean changes from baseline morning, afternoon, and evening CPRS-R ADHD index scores were -14.7 (10.90), -12.2 (12.89), and -13.4 (11.69) for the LDX groups, respectively, and -1.3 (14.92), -0.1 (9.01), and 0.4 (11.25) for the placebo group, respectively. These data were similar to the CPRS-R ADHD index scores observed in the overall population [17].

### Safety and Tolerability

Full safety analyses have been reported previously [17]. In the safety population, 196/290 (68%) participants reported one or more TEAEs; 21/290 (7.2%) discontinued due to TEAE. TEAEs with an incidence ≥ 5% in the combined LDX group were decreased appetite, insomnia, headache, upper abdominal pain, irritability, weight loss, vomiting, nausea, dizziness, and nasopharyngitis and, in the placebo group, were headache, cough, nasal congestion, nasopharyngitis, and upper abdominal pain. No serious AEs were observed during the study. More than 95% of TEAEs were mild or moderate in intensity and most began during the first week of treatment and abated over time [17]. Mean (SE) change from baseline at endpoint for pulse (bpm) ranged from 0.3 (1.20) to 4.1 (1.17) in all LDX groups and was -0.7 (1.17) in the placebo group. The systolic BP change for all LDX groups ranged from 0.4 (1.08) to 2.6 (1.05) mm Hg and for placebo was 1.3 (1.05) mm Hg. For diastolic BP the change ranged from 0.6 (0.93) to 2.3 (0.91) mm Hg for all LDX groups and was 0.6 (0.91) mm Hg for the placebo group. LDX treatment was not associated with any significant changes in mean BP, ECG parameters, and laboratory values.

## Discussion

In this post hoc analysis, LDX showed efficacy when given to children with significant clinical ADHD symptoms despite prior MPH treatment. Efficacy outcomes were similar to the results of the overall population assessed in the clinical trial.

Among participants previously treated with MPH, more than half were receiving doses (average daily dose ≥ 1 mg/kg) considered generally effective according to the regimens administered in randomized, controlled trials [28,29]. Conversely, just under half may have received suboptimal doses. Moreover, none of these measures differed from those observed in the overall study population. Although this study was not powered to detect differences between the treatment groups, the percentage of clinical responders in the overall study group was comparable regardless of LDX dose received. Similarly, no apparent differences occurred between the NNTs to achieve clinical response or symptomatic remission for the overall efficacy population and nonremitters on prior MPH. The NNT values calculated are comparable or superior to those reported elsewhere in the literature for symptomatic remission and clinical response to MPH and atomoxetine, which range from approximately 1.9 to 5.3 depending on formulation and types of raters [30].

Differential responses to MPH and amphetamine may explain a successful clinical response to LDX in participants who had significant ADHD symptoms despite prior MPH therapy. In 2 separate crossover studies [31,32] comparing the efficacy of MPH and dextroamphetamine, most children with ADHD who did not respond to 1 stimulant responded to the other. A bimodal pattern of clinical response to atomoxetine has been described, with no obvious demographic or clinical predictors of clinical response [33].

Clinical trial design may have contributed to the observed clinical response to LDX treatment in nonremitters on prior MPH. LDX treatment was administered in a forced-dose titration, while prior MPH therapy was provided according to community standards and included potential suboptimal dosing. Use of different definitions of therapeutic response may have altered the rates observed.

This post hoc analysis has limitations. The classification of participants as nonremitters on prior MPH considers only the ADHD-RS-IV total score at screening and may not reflect the participants' overall clinical response to MPH. It should be noted that switching from MPH formulations to LDX was done as part of the study protocol and not purely as a clinical practice decision.

The 4-week study duration limits the ability to extrapolate the findings to the long-term treatment generally required in managing ADHD. This study was not prospectively designed or powered to detect differences between the treatment groups. A prospective study would be required to confirm these preliminary findings.

## Conclusions

In this post hoc analysis of children who had significant clinical ADHD symptoms despite previous MPH treatment, LDX demonstrated efficacy and clinical response in the subpopulation assessed. Efficacy outcomes in this population were similar to those in the overall study population.

## List of Abbreviations

ADHD: attention-deficit/hyperactivity disorder; ADHD-RS-IV: ADHD Rating Scale IV; AE: adverse event; BP: blood pressure; CADDRA: Canadian ADHD Resource Alliance; CD: controlled delivery; CGI-S: Clinical Global Impressions-Severity; CGI-I: Clinical Global Impressions-Improvement; CI: confidence interval; CPRS-R: Short Form: Conners' Parent Rating Scale-Revised; dMPH: dexmethylphenidate; DSM-IV-TR*: Diagnostic and Statistical Manual of Mental Disorders*, *Fourth Edition, Text Revision*; ECG: electrocardiogram; ER: extended-release; IR: immediate-release; LDX: lisdexamfetamine dimesylate; MAS: mixed amphetamine salts; MPH: methylphenidate; NNT: number-needed-to-treat; OROS: osmotic release oral system; RCT: randomized controlled trial; SR: sustained-release; TEAE: treatment-emergent AE

## Competing interests

Dr Jain or Saundra Jain receives or has received grant research support from Abbott, Addrenex, Aspect, Forest, Lilly, and Pfizer; served as a consultant for Addrenex, Impax, Lilly, and Shire; served on a speaker's bureau for Cyberonics, GlaxoSmithKline, Jazz, Pfizer, Shire, and Takeda; received honorarium from Cyberonics, Forest, Jazz, Lilly, Pfizer, Roche, Shire, and Takeda. Dr Babcock is formerly an employee of Shire and holds stock and/or stock options in Shire. Dr Burtea is an employee of Shire Canada Inc. and holds stock and/or stock options in Shire Canada Inc. Dr Dirks is an employee of Shire and holds stock and/or stock options in Johnson & Johnson and Shire. Mr Adeyi is an employee of Shire and holds stock and/or stock options in Shire. Dr Scheckner is an employee of Shire and holds stock and/or stock options in Shire. Dr Lasser is an employee of Shire and holds stock and/or stock options in Shire.

## Authors' contributions

RJwas an investigator on the parent study and participated in data acquisition, analysis, interpretation, and presentation. RJ was fully involved in drafting the manuscript and revising the intellectual content of this manuscript. He has given final approval of this version. TBabcockwas the associate director, Scientific Publications, Clinical Development, and Medical Affairs for this study, and made substantial contributions to the analysis and interpretation of the data. He was deeply involved in drafting the manuscript and revising the intellectual content. He has given final approval of this version. TBurteawas the medical director, Global Clinical Development and Medical Affairs for this study and made substantial contributions to the analysis, and interpretation of the data. He was deeply involved in drafting the manuscript and revising the intellectual content. He has given final approval of this version. BDwas the director, Clinical Development and Medical Affairs for this study, and made substantial contributions to the analysis and interpretation of the data. He was deeply involved in drafting the manuscript and revising the intellectual content. He has given final approval of this version. BAwas a statistician involved in all post hoc data analysis, interpretation, and presentation. Statistician BA was fully involved in drafting and revising the intellectual content of this manuscript. Statistician BA has given final approval to this version. BSwas the associate director, Scientific Publications, Clinical Development, and Medical Affairs for this study, and made substantial contributions to the analysis and interpretation of the data. He was deeply involved in drafting the manuscript and revising the intellectual content. He has given final approval of this version. RLwas the senior director, Clinical Development and Medical Affairs for this study, and made substantial contributions to the analysis and interpretation of the data. He was deeply involved in drafting the manuscript and revising the intellectual content. He has given final approval of this version.
